# Mediating EGFR-TKI Resistance by VEGF/VEGFR Autocrine Pathway in Non-Small Cell Lung Cancer

**DOI:** 10.3390/cells11101694

**Published:** 2022-05-19

**Authors:** Chike Osude, Leo Lin, Meet Patel, Adam Eckburg, Joseph Berei, Adijan Kuckovic, Namrata Dube, Aayush Rastogi, Shruti Gautam, Thomas J. Smith, Shylendra B. Sreenivassappa, Neelu Puri

**Affiliations:** 1 Department of Biomedical Sciences, University of Illinois College of Medicine at Rockford, Rockford, IL 61107, USA; cosude@bluebirdbio.com (C.O.); llin9@northwell.edu (L.L.); mpate307@uic.edu (M.P.); adam-eckburg@northwestern.edu (A.E.); jberei2@uic.edu (J.B.); akuckovic@arrowheadpharma.com (A.K.); ndube3@uic.edu (N.D.); arasto5@uic.edu (A.R.); sgauta3@uic.edu (S.G.); 2 College of Education, Northern Illinois University, Dekalb, IL 60115, USA; tjsmith@niu.edu; 3 Department of Hematology/Oncology, OSF Saint Anthony Medical Center, Rockford, IL 61107, USA; shylendra.b.sreenivasappa@osfhealthcare.org

**Keywords:** non-small cell lung cancer (NSCLC), tyrosine kinase inhibitor (TKI), epidermal growth factor receptor (EGFR), vascular endothelial growth factor receptor-2 (VEGFR-2), vascular endothelial growth factor (VEGF), neuropilin-1 (NP-1), tumors, survival analysis

## Abstract

**Simple Summary:**

Non-small cell lung cancer (NSCLC) patients acquire resistance to tyrosine kinase inhibitors (TKIs) via EGFR mutations or overexpression of vascular endothelial growth factor receptor-2 (VEGFR-2). In this study, we elucidated the mechanism of EGFR-TKI resistance mediated by VEGF/VEGFR in EGFR-mutated NSCLC cell lines and Erlotinib-resistant cell lines as compared to parental cell lines. Increased expression of VEGF, VEGFR-2, and NP1 was observed in Erlotinib-resistant cell lines. Furthermore, we observed an increased efficacy of Erlotinib in combination with a VEGFR-2 inhibitor in Erlotinib-resistant cell lines. Late-stage NSCLC patients with high expression of VEGFR-2 had shorter survival times compared to patients with low VEGFR-2 expression. These results indicate that VEGFR-2 may play a key role in EGFR-TKI resistance that can be overcome with a combination treatment of Erlotinib and a VEGFR-2 inhibitor, which may serve as an effective treatment option for NSCLC patients with EGFR mutations.

**Abstract:**

NSCLC treatment includes targeting of EGFR with tyrosine kinase inhibitors (TKIs) such as Erlotinib; however, resistance to TKIs is commonly acquired through T790M EGFR mutations or overexpression of vascular endothelial growth factor receptor-2 (VEGFR-2). We investigated the mechanisms of EGFR-TKI resistance in NSCLC cell lines with EGFR mutations or acquired resistance to Erlotinib. These studies showed upregulated gene and protein expression of VEGF, VEGFR-2, and a VEGF co-receptor neuropilin-1 (NP-1) in Erlotinib-resistant (1.4–5.3-fold) and EGFR double-mutant (L858R and T790M; 4.1–8.3-fold) NSCLC cells compared to parental and EGFR single-mutant (L858R) NSCLC cell lines, respectively. Immunofluorescence and FACS analysis revealed increased expression of VEGFR-2 and NP-1 in EGFR-TKI-resistant cell lines compared to TKI-sensitive cell lines. Cell proliferation assays showed that treatment with a VEGFR-2 inhibitor combined with Erlotinib lowered cell survival in EGFR double-mutant NSCLC cells to 9% compared to 72% after treatment with Erlotinib alone. Furthermore, Kaplan–Meier analysis revealed shorter median survival in late-stage NSCLC patients with high vs. low VEGFR-2 expression (14 mos vs. 21 mos). The results indicate that VEGFR-2 may play a key role in EGFR-TKI resistance and that combined treatment of Erlotinib with a VEGFR-2 inhibitor may serve as an effective therapy in NSCLC patients with EGFR mutations.

## 1. Introduction

Lung cancer is the leading cause of cancer-related deaths. It was projected that, in 2021, 235,760 new lung cancer cases would be diagnosed and that 131,880 individuals would die from the disease in the United States alone [1]. Non-small cell lung carcinoma (NSCLC), the most prevalent form of the disease, is more likely to be detected in its later stages (IIIb-IV) than other forms of cancer because early-stage NSCLC patients are often asymptomatic [2,3]. As a result of lung cancer’s high mortality rate, 30% of NSCLC patients die within 90 days of diagnosis [3]. The low survival rate of NSCLC has led to many studies focusing on molecular targets with therapeutic potential for treatment of the disease [4]. Among these targets, epidermal growth factor receptor (EGFR) has been studied most extensively.

EGFR is a receptor tyrosine kinase (RTK) overexpressed in 40–80% of NSCLC patients. When dysregulated, EGFR facilitates tumorigenic signaling [5,6]. Expression of EGFR in NSCLC patients is correlated with frequent lymph node metastasis, poor chemo-sensitivity, and a decrease in overall survival [7]. EGFR tyrosine kinase activity can be dysregulated through mutations in the EGFR gene, increased copy number, and protein expression in NSCLC [8]. Upon activation of the EGFR family, the RAS/RAF/MEK/MAPK and PI3K/AKT/m-TOR pathways are activated, leading to increased cell proliferation, cell growth, invasion, metastasis, apoptosis, and tumor angiogenesis [6,9].

Numerous tyrosine kinase inhibitors (TKIs) have been developed to act against activating mutations in EGFR. First-generation TKIs such as Erlotinib and second-generation TKIs such as Afatinib are used to overcome EGFR aberrant signaling by covalently binding to EGFR’s kinase domain [4]. The development of TKI resistance, however, is common after only 9–14 months of treatment [10,11]. Fifty percent of cases with acquired resistance to EGFR-TKI treatment are due to the development of a T790M mutation in the EGFR kinase domain [12]. This secondary mutation occurs in the presence of a primary L858R mutation and results in a large methionine residue being substituted for a threonine at position 790 of exon 20 [4,13]. This change drastically diminishes the binding effect of TKIs and increases the affinity of ATP for its binding pocket [14].

As observed in lung cancer, dysregulation of the EGFR pathway results in upregulation of the human vascular endothelial growth factor (VEGF) pathway. Previous studies using cell culture models have shown that EGFR activation via epidermal growth factor (EGF) or transforming growth factor alpha (TGF-α) increases the production of VEGF [15,16]. Vascular endothelial growth factor receptor 2 (VEGFR-2) is a protein mainly expressed on the surface of endothelial cells and it serves as the primary mediator of VEGF signaling and vascular angiogenesis [17,18,19,20]. When VEGF binds to VEGFR-2, it causes receptor dimerization and activation of downstream intracellular signaling cascades, such as the MAPK, mTOR, and FAK-Paxillin pathways [18,21]. These pathways promote angiogenesis by contributing to the survival, proliferation, and migration of endothelial cells [18]. Interestingly, it has been confirmed that the VEGFRs can also be expressed on the surface of tumor cells, including those associated with NSCLC [22,23,24]. In NSCLC, an autocrine feed-forward loop has been discovered in which tumor-derived VEGF stimulates an increase in VEGF production via VEGFR-2-dependent activation of the mTOR pathway, substantially amplifying the initial proangiogenic signal [25]. Hypoxia and aberrant growth factor signaling in NSCLC tumors are two factors primarily responsible for VEGF overproduction and the resultant poor prognosis in NSCLC [26,27,28]. The interconnection between the VEGF and EGFR pathways has created the rationale for their dual inhibition as a potential therapy, especially in NSCLC cases [15,16,29].

Neuropilins (NPs) (also commonly known as NRP-1s) are non-tyrosine kinase receptors that act as VEGF co-receptors. Prior studies have shown that co-expression of NPs is significantly correlated with poor prognosis and tumor progression in NSCLC [30]. Neuropilin-1 (NP-1) possesses a small intracytoplasmic domain that is believed to play a key role in the receptor’s angiogenic activity [31]. Preclinical studies have demonstrated that NP-1 plays a role in lung cancer cell migration, proliferation, and invasion and also results in poor prognosis [30,32]. NPs alone do not have internal catalytic activity, but they control the endocytosis, intracellular trafficking, and downstream signaling of VEGFR-2. NP-1 forms complexes with VEGFR-1 and VEGFR-2 to greatly enhance the binding of specific isoforms of VEGF to VEGFRs, increasing tumorigenicity and neovascularization [33,34].

To better understand the mechanism of EGFR-TKI resistance in NSCLC and to develop more effective therapies against it, we studied three biomarkers linked to increased angiogenesis in NSCLC [18]. We investigated whether an autocrine feed-forward mechanism leads to upregulation of VEGF, VEGFR-2, and NP-1 in EGFR-TKI-resistant cell lines and results in angiogenesis, tumorigenicity, and NSCLC progression. Although many studies have been dedicated to EGFR and VEGF inhibition, there has been a minimal increase reported in the overall survival of lung cancer patients [35]. Several studies have focused on the role of VEGF and VEGF inhibitors in acquired resistance to EGFR TKIs. In this study we have found that VEGF secretion was not increased in Erlotinib-resistant cells compared to parental cells. We found that VEGFR2 plays a key role in EGFR TKI resistance and that VEGFR2 inhibitors can be a better alternative to increase the overall survival of NSCLC patients. In this study, the main focus is to develop a better treatment alternative by targeting VEGFR in combination with an EGFR inhibitor, erlotinib, which has not been extensively studied and which could be used as part of a future approach. Our studies explored the role of VEGFR-2 in the prognosis of NSCLC and whether modulation of VEGF/VEGFR-2 may help overcome resistance to TKIs. VEGFR-2 was found to play a key role in EGFR-TKI resistance and the combinatory use of Erlotinib with a VEGFR-2 inhibitor showed therapeutic promise for NSCLC patients with EGFR mutations.

## 2. Materials and Methods

### 2.1. The Tyrosine Kinase Inhibitors and Growth Factor Ligands

Erlotinib hydrochloride (N-(3-ethynylphenyl)-6,7-bis(2-methoxyethoxy) quinazolin-4-amine; C22H23N3O4•HCl) was obtained from LC laboratories (Woburn, MA, USA), reconstituted in Dimethyl Sulfoxide (DMSO) at a concentration of 20 mM, and then stored in aliquots of 20 µL at −20 °C. ZM 323,881 hydrochloride (5-((7-Benzyloxyquinazolin-4-yl)amino)-4-fluoro-2-methylphenol hydrochloride), a selective inhibitor of VEGFR2, was obtained from Tocris Bioscience, a Bio-Techne brand (Minneapolis, MN, USA) [36], reconstituted in DMSO at a concentration of 1 mM, and then stored in aliquots at −20 °C. Epidermal Growth Factor (EGF) was obtained from Peprotech (Rocky Hill, NJ, USA), reconstituted in PBS to obtain a concentration of 15 ng/µL, and aliquots were stored at −20 °C.

### 2.2. Antibodies

Rabbit polyclonal antibody for VEGFR-2 (cat. no. SAB4501645-100UG) was obtained from Sigma-Aldrich. Rabbit polyclonal antibody for VEGFR-2 (cat. no. 07-716-I) was obtained from Millipore (Billerica, MA, USA). Rabbit mouse monoclonal neuropilin-1 antibody (cat. no. Ab81321) was obtained from Abcam (Cambridge, MA, USA). Goat polyclonal antibody for neuropilin-1 (cat. no. sc-7239) was obtained from Santa Cruz (Dallas, TX, USA). Mouse monoclonal VEGF (C-1) antibody (cat. no. sc-7269) was obtained from Santa Cruz (Dallas, TX, USA). Bevacizumab, a humanized monoclonal VEGF-165 antibody (cat. no. NBP2-59637), was obtained from Novus Biologicals (Littleton, CO, USA). Human antibodies CD309 (VEGFR-2/KDR)-APC (cat. no. 130-098-910) and CD304-PE (BDCA-4/Neuropilin-1) (cat. no. 130-098-876) were obtained from MACS Miltenyi Biotec (Cambridge, MA, USA). All primary antibodies were diluted according to the manufacturer’s instructions in TBST with 1% BSA. Secondary antibodies were prepared at a dilution of 1:1000 in TBST with 1% blocking grade milk (cat. no. 170-6404XTU) from Bio-Rad (Hercules, CA, USA).

### 2.3. Cell Lines

H2170, H358, H3255, and H1975 NSCLC cell lines (cat. nos. CRL-5928, CRL-5807, CRL-2882, and CRL-5908, respectively) were purchased from American Type Culture Collection (ATCC) (Rockville, MD, USA). Cell lines were grown in incubators at 37 °C with 6% CO_2_ and cultured according to ATCC’s instructions. H2170 and H358 cell lines have wild-type EGFR status, whereas H3255 (L858R) and H1975 (L858R and T790M) cell lines have mutated EGFR, as documented by ATCC. H2170 and H358 cell lines were cultured in increasing drug concentrations of Erlotinib to obtain EGFR-TKI-resistant cells. H2170-ER and H358-ER cells were obtained and cultured in the presence of erlotinib, as described previously [37]. The doubling time of these cells is shown in Table S1 in the Supplementary Materials.

### 2.4. Quantitative Real-Time PCR

H2170 parental and erlotinib-resistant (P/ER) and H358-P/ER cells were plated in 35 mm petri dishes and allowed to adhere and grow for 24 h. Cells were then starved for 24 h and total RNA was collected using a PureLink RNA Mini Kit (cat. no. 12183018A) from Life Technologies, according to the manufacturer’s protocol. RNA was quantified using the Take3 NanoDrop method. Total RNA was used to perform qPCR with an Applied Biosystems 7300 Real Time-PCR system. For this procedure, a SuperScript™ III Platinum™ SYBR™ Green One-Step qRT-PCR Kit (cat. no. 11736059) from Life Technologies was employed and the manufacturer’s protocol was followed. The cycle threshold (Ct) values were recorded and normalized using GAPDH, and 2^(−Δ(ΔCt))^ was calculated to evaluate the fold changes in gene expression. All primers (Table 1) used for qPCR were purchased from Integrated DNA Technologies (Coralville, IA, USA).

### 2.5. Immunoblotting

NSCLC cells were cultured as described previously [38]. H2170-ER and H358-ER cells were then treated with TKIs (10 μM erlotinib in serum-free RPMI media with 0.5% BSA for 24 h) and subsequently with ligands (15 ng/mL EGF for 2.5 min or 40 ng/mL HGF for 7.5 min in serum-free media). Cell lysates were prepared, electrophoresed, and transferred onto nitrocellulose membranes, as described previously [38]. Membranes were probed with antibodies for VEGFR-2 and NP-1. Immunoblots were developed using a Pierce ECL Substrate Chemiluminescence Kit (Thermo Fisher Scientific, Waltham, MA, USA), and modulations in protein expression were calculated by densitometric analysis using NIH ImageJ software.

### 2.6. Immunofluorescence

NSCLC cells were seeded on an eight-well chamber slide, fixed, permeabilized, blocked, and incubated at 4 °C overnight with primary antibodies for VEGFR-2 and NP-1. Primary antibodies were then removed and the cells were incubated in the dark at room temperature for an hour with anti-mouse/rabbit IgG secondary antibody conjugated with DyLight^TM^ 488 (1:250) (Thermo Fisher Scientific, Waltham, MA, USA, cat. no. 35502) and Hoechst nuclear staining dye (1:10,000) (Life Technologies, Carlsbad, CA, USA, cat. no. 33342) diluted in 1X PBS with 1% BSA. Slides were then mounted and covered with a coverslip using fluorogel (Electron Microscopy Sciences, Hatfield, PA, USA, cat. no. 17985-10). Stained cells were observed using an Olympus Fv10i Fluoview Confocal Microscope and images were captured. Average fluorescence intensity of staining was quantitatively measured using Olympus Fluoview image analysis software.

### 2.7. Flow Cytometry

NSCLC cells were seeded in a 100 mm petri dish. After 48 h, the growth medium was removed. The cells were then washed with PBS and treated with Accutase Cell Detachment Solution (Affymetrix, eBioscience, San Diego, CA, USA, cat. no. 00-4555-56) in an incubator for five minutes or until all of the cells were detached. Cells were collected in a 15 mL tube, pelleted using a centrifuge, and then re-suspended in flow cytometry buffer (PBS + 2 mM EDTA + 0.5% BSA). Cells were pelleted again and probed with fluorophore-labeled antibody for ten minutes on ice. The cells were washed with flow cytometry buffer to remove excess antibody, then suspended in FACS buffer for analysis with a BD FACS CALIBUR flow cytometer.

### 2.8. MTT Cell Viability Assay

NSCLC cells were seeded on a 96-well plate in RPMI medium with 10% FBS. After 24 h, cells were treated with TKIs, suspended in 100 µL starving media (RPMI + 0.5% BSA) per well, and incubated for an additional 72 h at 37 °C. Cells were then washed with RPMI media without phenol red and 100 µL of phenol red-free RPMI media was added to the cells. Subsequently, 10 µL of MTT reagent (Thiazolyl Blue Tetrazolium Bromide Dye; Sigma-Aldrich, St. Louis, MO, USA, cat. no. M5655) was added to each well and plates were incubated for four hours at 37 °C. Finally, 100 µL of MTT solubilization reagent (acidic isopropanol) was added to each well and mixed to dissolve any and all formazan crystals formed. Absorbance was measured using a spectrophotometer at wavelengths of 570 nm and 630 nm, and percent cell viability was calculated with respect to controls. This experiment was performed in replicates of six for each treatment condition.

### 2.9. ELISA

NSCLC cells were plated in 25 cm^2^ vented flasks and allowed to adhere for 24 h in RPMI medium with 10% FBS. Medium was then removed, followed by two 1X PBS washes. Serum-free medium was added and incubated for 24 h. Then, the medium was pipetted into a micro-centrifuge tube and centrifuged for ten minutes at 4 °C with a LABNET Refrigerated Z326K centrifuge (HERMLE Labortechnik, Wehingen, Germany) at 1500 rpm. The supernatant was immediately aliquoted, avoiding the debris pelleted, and samples were stored at −70 °C. Collection of conditioned medium was repeated for 24 and 48 h time points. The commercial ELISA kit Human VEGF-A ELISA from RayBiotech (Nor-cross, GA, USA) was used to detect and quantify the amount of VEGF in the NSCLC cell lines at two separate time points: 24 h and 48 h. The vendor’s protocol was followed to run the experiment, and each test was run in triplicate.

### 2.10. Immunohistochemistry

We analyzed 79 paraffin-embedded, formalin-fixed tissues from patients with NSCLC. All cases were retrieved from the pathology archives at Rockford Memorial Hospital and the Swedish American Hospital with institutional approval, in accordance with the Institutional Review Board protocol (351597-11 and 17 August 2018). Immunostaining procedures were conducted as described previously [39]. Appropriate negative controls were prepared by omitting the primary antibody step and substituting it with non-immune rabbit serum. Normal healthy controls for immunostaining were prepared as well. Lung cancer patients diagnosed at stages 1–3A are classified as early-stage and patients diagnosed at stages 3B-4 are classified as late-stage.

### 2.11. Statistical Analysis

All experiments were performed three to five times. The Student’s *t*-test was used to analyze the statistical significance of the data. A *p*-value of less than 0.05 was considered statistically significant throughout the study.

## 3. Results

### 3.1. Increased Gene Expression of VEGF, VEGFR-2, and NP-1 in Erlotinib-Resistant NSCLC Cells

**Figure 1:**
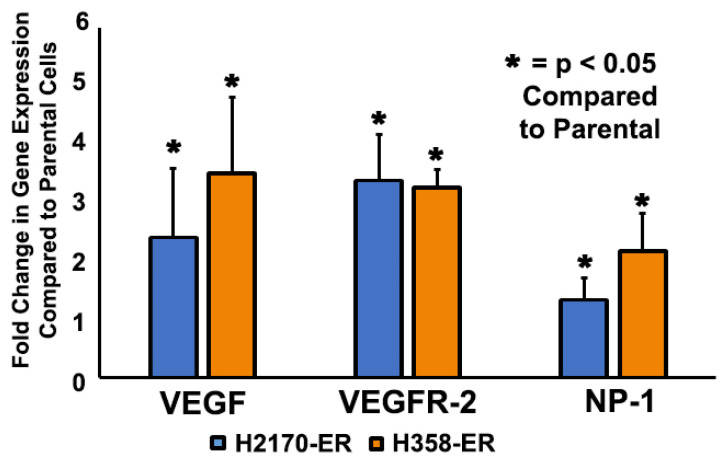
**Increased gene expressions of VEGF, VEGFR-2, and NP-1 in Erlotinib-resistant NSCLC cell lines.** For the analysis, 2.5 × 10^5^ H358-P/ER and H2170-P/ER cells were plated in Petri dishes and allowed to adhere and grow for 24 h. The cells were then starved (RPMI + 0.5% BSA) for 24 h and total RNA was collected. Real-time quantitative polymerase chain reaction (qPCR) was then performed with three duplicates for each target gene as well as with a housekeeping gene (GAPDH). mRNA was quantified and analyzed for VEGF, VEGFR-2, and NP-1. The data was normalized with GAPDH and the graphical representation is relative to expression of the respective genes in H2170-P and H358-P cells. Statistical analyses demonstrated that the results were statistically significant (*p* < 0.05) by way of two-tailed *t*-test analyses.

The gene expressions of potential EGFR-TKI-resistance modulators were analyzed in the wild type-EGFR NSCLC cell lines H358-P and H2170-P and compared to corresponding Erlotinib-resistant H358-ER and H2170-ER cell lines. H358 cell lines also possess a unique KRAS mutation. The results demonstrated an increase in gene expression of VEGF (2.5-fold), VEGFR-2 (3.5-fold), and NP-1 (1.4-fold) in H2170-ER compared to H2170-P cells, as can be seen in Figure 1. Additionally, the results demonstrated an increase in gene expressions of VEGF (3.6-fold), VEGFR-2 (3.3-fold), and NP-1 (2.2-fold) in H358-ER compared to H358-P cells (Figure 1).

### 3.2. Modulation of Protein Expression of VEGFR-2 and NP-1 in NSCLC Cell Lines

**Figure 2:**
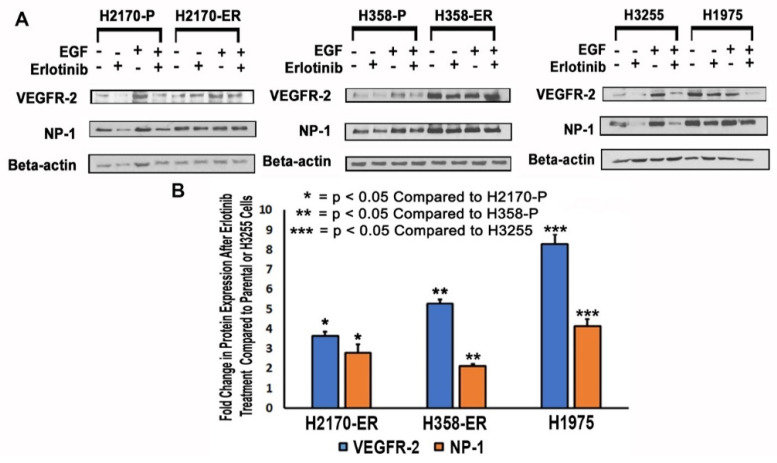
**Modulation of VEGFR-2 and NP-1 protein expression in NSCLC cell lines**. (**A**) For the analysis, 2.5 × 10^5^ H2170-P/ER, H358-P/ER, H1975, and H3255 cells were seeded in Petri-dishes. Cells were allowed to adhere and grow for 24 h, then starving media (RPMI with 0.5% BSA) was added for 24 h. Cells were then treated with Erlotinib (10 µM Erlotinib) for 24 h and/or with ligands (15 ng/mL EGF for 2.5 min in starving media) before collection of cell lysates. Immunoblotting was then performed. (**B**) The data were normalized with beta-actin, and the graphical representation is relative to protein expression of the corresponding parental or single-mutant cell line. The modulations were calculated by densitometric analysis using ImageJ software. Statistical analysis was performed and it was demonstrated that the results were statistically significant (*p* < 0.05) by way of a two-tailed *t*-test analysis.

Immunoblotting studies were performed to assess the modulation of VEGFR-2 and NP-1 protein expression in wild-type EGFR NSCLC cells (H2170-P/ER and H358-P/ER), TKI-sensitive EGFR L858R-mutant NSCLC cells (H3255), and TKI-resistant EGFR L858R- and T790M-mutant NSCLC cells (H1975). Each cell line was analyzed without treatment, after treatment with Erlotinib, after treatment with epidermal growth factor (EGF), and after treatment with a combination of Erlotinib and EGF (Figure 2A).

The expressions of VEGFR-2 and NP-1 from Western blots were quantified and analyzed. The results demonstrated an increase in VEGFR-2 (3.6-fold) and NP-1 (2.8-fold) expression in the presence of 10 µM Erlotinib in H2170-ER cells compared to the H2170-P cell line subjected to the same treatment (Figure 2B). Additionally, in H358-ER cells as compared to the parental line, there was an increase in protein expression of VEGFR-2 (5.3-fold) and NP-1 (2.1-fold) in the presence of 10 µM Erlotinib (Figure 2B). The results also demonstrated increased levels of VEGFR-2 (8.3-fold) and NP-1 (4.1-fold) in the presence of 10 µM Erlotinib in H1975 cells as compared to H3255 cells (Figure 2B).

### 3.3. Increased Cell Surface Expression of VEGFR-2 and NP-1 Receptors in Erlotinib-Resistant NSCLC Cell Lines, as Demonstrated by FACS Analysis

**Figure 3:**
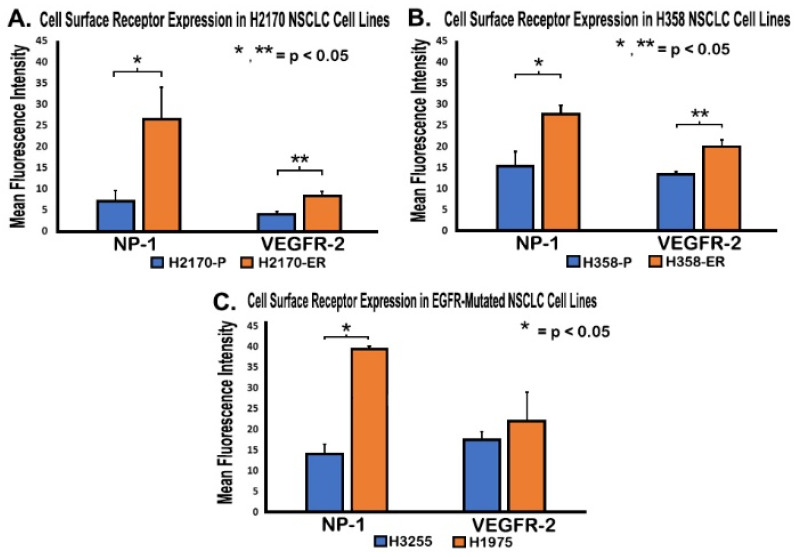
**Increased cell surface expression of VEGFR-2 and NP-1 receptors in Erlotinib-resistant NSCLC cell lines.** For the analysis, 5 × 10^5^ H2170-P/ER, H358-P/ER, H1975, and H3255 cells were seeded in a Petri dish and allowed to grow and adhere for 48 h. After the growth medium was removed, the cells were washed and detached. Cells were then collected, pelleted, re-suspended in a buffer, pelleted again, probed with antibody, washed, and re-suspended in buffer before being used for flow cytometry. Cell surface expression was compared in (**A**) H2170-ER cells compared to H2170-P cells, **(B**) H358-ER cells compared to parental cells, and (**C**) T790M- and L858R-mutant H1975 cells compared to L858R-mutant H3255 cells. Statistical analyses were performed, and it was demonstrated that the results were statistically significant (*p* < 0.05) by m of two-tailed *t*-test analyses. One asterisk (*) denotes the statistical significance of data concerning NP-1, whereas two asterisks (**) denote the statistical significance of data concerning VEGFR-2.

After observing upregulation of gene and protein expression of VEGFR-2 and NP-1 in ER cells, the levels of expression of these proteins were measured on cell surfaces. Flow cytometry assays were performed to measure the expression of VEGFR-2 and NP-1 in H2170-P/ER, H358-P/ER, H3255, and H1975 cell lines. In H2170-ER cells as compared to H2170-P cells, there was an increase in mean fluorescent intensity for VEGFR-2 (2.1-fold) and NP-1 (3.8-fold) (Figure 3A). In H358-ER cells as compared to H358-P cells, there was also an increase in mean fluorescent intensity of VEGFR-2 (1.5-fold) and NP-1 (1.8-fold) (Figure 3B). Additionally, the results showed an increase in mean fluorescent intensity in VEGFR-2 (1.3-fold) and NP-1 (2.8-fold) for H1975 cells when compared to H3255 cells (Figure 3C).

### 3.4. Increased Cell Surface Expression of VEGFR-2 and NP-1 Receptors in Erlotinib-Resistant NSCLC Cell Lines, as Demonstrated by Immunofluorescence Studies

**Figure 4:**
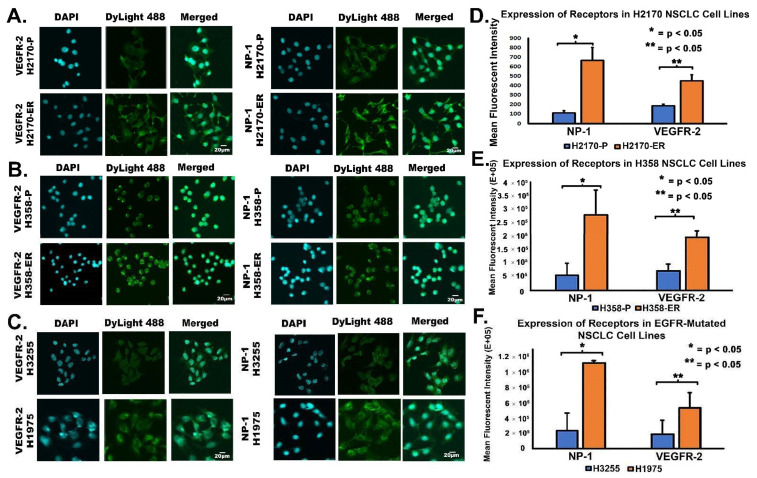
**Immunostaining and expression of VEGFR-2 and NP-1 in NSCLC cell lines.** Immunofluorescent images showing modulation in EGFR-TKI resistance-related proteins in (**A**) H2170-P/ER, (**B**) H358-P/ER, and (**C**) H1975 and H3255 cells. For the analysis, 2 × 10^4^ cells per well were plated in an 8-well chamber slide, fixed, permeabilized, and probed with anti-VEGFR-2 and anti-NP-1 primary antibodies and a DyLight 488 conjugated secondary antibody for immunostaining. Nuclei were stained using DAPI, and cells were visualized using an Olympus Fluoview confocal microscope. The mean fluorescent intensities of VEGFR-2 and NP-1 receptors were graphically represented for (**D**) H2170-ER cells as compared to parental cells, (**E**) H358-ER cells as compared to H358-P cells, and (**F**) H1975 double-mutants (T790M and L858R) as compared to H3255 single-mutant (L858R) cells. Fluorescence quantifications were performed using Olympus Fluoview image analysis software, and mean fluorescent intensities are graphically depicted. Statistical analyses demonstrated that the results were statistically significant (*p* < 0.05) by way of two-tailed *t*-test analyses. One asterisk (*) denotes the statistical significance of data concerning NP-1, whereas two asterisks (**) denote the statistical significance of data concerning VEGFR-2.

Immunofluorescence (IF) was performed to confirm and further study the specific localization of VEGFR-2 and NP-1 in parental (H2170-P and H358-P), Erlotinib-resistant (H2170-ER and H358-ER), EGFR single-mutated (H3255), and EGFR double-mutated (H1975) cell lines. In H2170-ER cells, VEGFR-2 was expressed mainly on the cell surface and in the cytoplasm, while in H2170-P cells, VEGFR-2 was expressed mainly in the cytoplasm (Figure 4A). In H2170-ER cells, NP-1 was expressed in the cytoplasm, on the cell surface, and in the nucleus, while in parental cells, NP-1 was minimally expressed (Figure 4A). Analysis of the fluorescent images revealed increased mean fluorescent activities of NP-1 (6.0-fold) and VEGFR-2 (2.4-fold) in H2170-ER as compared to H2170-P cells (Figure 4D).

In H358-ER cells, VEGFR-2 was expressed on the cell surface, in the cytoplasm, and in the nucleus, while in H358-P cells, VEGFR-2 was minimally expressed on the cell surface, in the nucleus, and in the cytoplasm (Figure 4B). NP-1 was highly expressed in the cytoplasm and on the cell surface of H358-ER cells, while NP-1 was only marginally expressed on the cell surface of H358-P cells (Figure 4B). There was an increase in the mean fluorescent intensity of NP-1 (5.1-fold) and VEGFR-2 (2.2-fold) in H358-ER cells compared to H358-P cells (Figure 4E).

In H1975 cells, VEGFR-2 was expressed on the cell surface, in the cytoplasm, and in the nucleus, while VEGFR-2 was minimally expressed in the nucleus and on the membrane of H3255 cells (Figure 4C). NP-1 was highly expressed on the membrane and in the cytoplasm of H1975 cells, but it was minimally expressed on the membrane and in the cytoplasm of H3255 cells (Figure 4C). The results also showed increased mean fluorescent intensities of NP-1 (4.2-fold) and VEGFR-2 (3.2-fold) in H1975 cells compared to H3255 cells (Figure 4F). 

### 3.5. Secretion of VEGF by NSCLC Cell Lines

**Figure 5:**
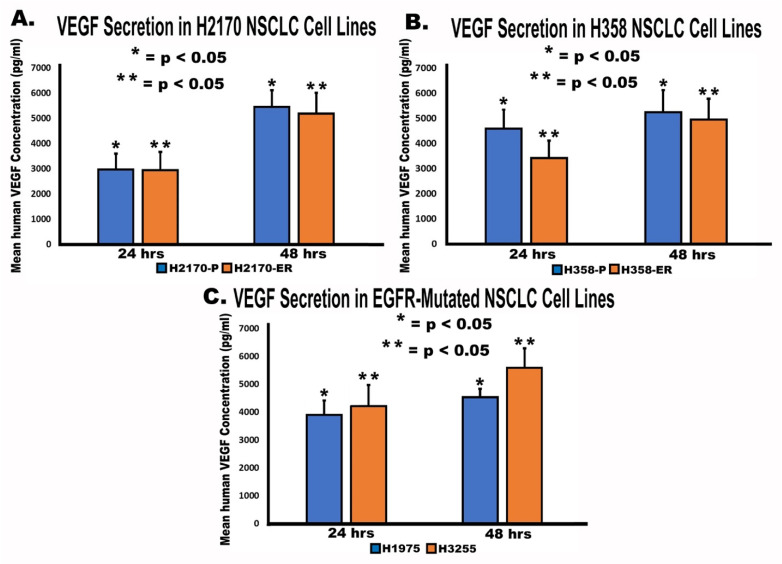
**Secretion of VEGF in NSCLC cell lines.** For the analysis 3.75 × 10^6^ H2170-P/ER, H358-P/ER, H1975, and H3255 cells were plated in vented flasks and allowed to adhere for 24 h in RPMI medium with 10% FBS. After 24 h, medium was removed, followed by PBS washes, and then starving medium (RPMI + 0.5% BSA) was added and incubated for 24 h. The medium was then centrifuged, and the pellets were stored in −70 °C. Collection of conditioned medium was repeated for 24 h and 48 h time points. The amount of VEGF in (**A**) H2170-P/ER, (**B**) H358-P/ER, and (**C**) H1975 and H3255 NSCLC cell lines was quantified at two time points, 24 and 48 h, and the experiments were run in triplicate. Statistical analysis was performed, and the results were found to be statistically significant (* = *p* < 0.05) by way of a two-tailed *t*-test analysis. One asterisk (*) denotes statistical significance in VEGF secretion at two different time points in parental (A, B) or EGFR double-mutant (C) cell lines, whereas two asterisks (**) denote statistical significance in VEGF secretion at two different time points in Erlotinib-resistant cell lines (A, B) or EGFR single-mutant cell lines.

An enzyme-linked immunosorbent assay (ELISA) was performed to study the role of VEGF secretion in angiogenesis and EGFR-TKI resistance. Human VEGF concentration was quantified and analyzed for all cell lines and used to calculate levels of VEGF secretion. The results demonstrated that VEGF concentration (pg/mL) increased from 24 h to 48 h in all cell lines studied (Figure 5). We observed increases in VEGF secretion of 84% and 77% between 24 and 48 h in H2170-P and H2170-ER cells, respectively (Figure 5). In H358-P and H358-ER cell lines, VEGF secretion increased by 14% and 45%, respectively, between 24 and 48 h (Figure 5). Finally, VEGF secretion increased by 33% in H3255 cells and by 16% in H1975 cells between 24 and 48 h (Figure 5). No significant difference was observed in the magnitudes of increase in human VEGF secretion between H2170-P and H2170-ER cell lines, between H358-P and H358-ER cell lines, nor between H3255 and H1975 cell lines.

### 3.6. Effect of Anti-VEGF Treatments on the Proliferation of Erlotinib-Resistant and EGFR-Mutated NSCLC Cell Lines

**Figure 6:**
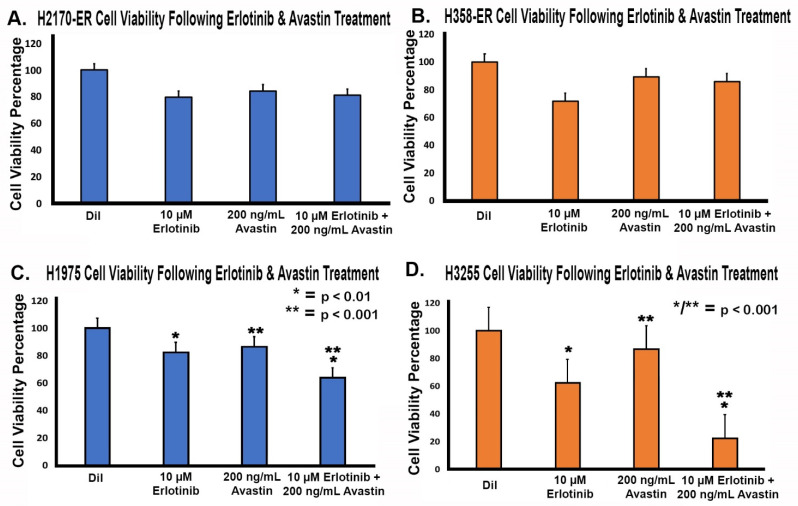
**Effect of anti-VEGF treatment in NSCLC cell lines.** A total of 8,000 H2170-ER, H358-ER, H1975, and H3255 NSCLC cells were treated with diluent (control), 10 µM Erlotinib treatment, 200 ng/mL Avastin treatment, and a combination of 10 µM Erlotinib and 200 ng/mL Avastin. Cell viability was assessed with an MTT cell viability assay to study the efficacy of Erlotinib in conjunction with the VEGF inhibitor, Avastin, relative to the control. In (**A**) H2170-ER cells, (**B**) H358-ER cells, (**C**) H1975 cells, and (**D**) H3255 cells, cell viability was measured. Six duplicates from each cell line were used for each treatment group. Absorbance was recorded, and percent viability was calculated using appropriate controls for each sample. Statistical analysis was performed, and the results were found to be statistically significant (* = *p* < 0.05) by way of two-tailed *t*-test analysis. All treatment groups showed statistically significant results compared to the diluent. Treatment groups without an asterisk (*) did not show statistically significant results. One asterisk (*) denotes statistical significance in all the bars with one asterisk and two asterisks (**) suggest statistical significance between all the bars with two asterisks.

To further study the effect of VEGF in EGFR-TKI resistance, we investigated the use of a humanized monoclonal VEGF antibody, bevacizumab (Avastin), which binds to and neutralizes VEGF. An MTT cell viability assay was performed to assess the effects of Avastin, Erlotinib, and both in combination in Erlotinib-resistant and EGFR-mutated NSCLC cells lines. Cells being resistant to 10 µM of Erlotinib, this was the concentration that was used, and since Avastin showed a similar inhibitory effect on cells in a concentration range of 200 ng/mL to 1000 ng/mL, we used 200 ng/mL of Avastin. The 10 µM Erlotinib treatment decreased cell viability to between 62 and 82% compared to the diluent, while the 200 ng/mL Avastin treatment decreased cell viability to between 84 and 89% compared to the diluent (Figure 6). Furthermore, the combination treatment of 10 µM Erlotinib and 200 ng/mL Avastin decreased cell viability to between 22% and 85% when compared to the diluent (Figure 6). Our results suggested that Avastin had minimal anti-proliferative effect. A minimal decrease in cell viability was observed in cells treated with Avastin alone compared to cells treated with Erlotinib alone. In addition, there was no statistically significant decrease in cell viability in H2170-ER and H358-ER cells treated with a combination of Avastin and Erlotinib as compared to Erlotinib and Avastin alone, whereas in both H1975 and H3255 cells there was a significant decrease in cell viability with the combination treatment of 10 µM Erlotinib and 200 ng/mL Avastin as compared to Erlotinib and Avastin alone. Furthermore, the combination treatment of 10 µM Erlotinib and 200 ng/mL Avastin in double-mutant H1975 cells showed an increase in cell viability to 64% from 22% in single-mutant H3255 cells.

### 3.7. Effect of Anti-VEGFR-2 Treatments in Erlotinib-Resistant and EGFR-Mutated NSCLC Cell Lines

**Figure 7:**
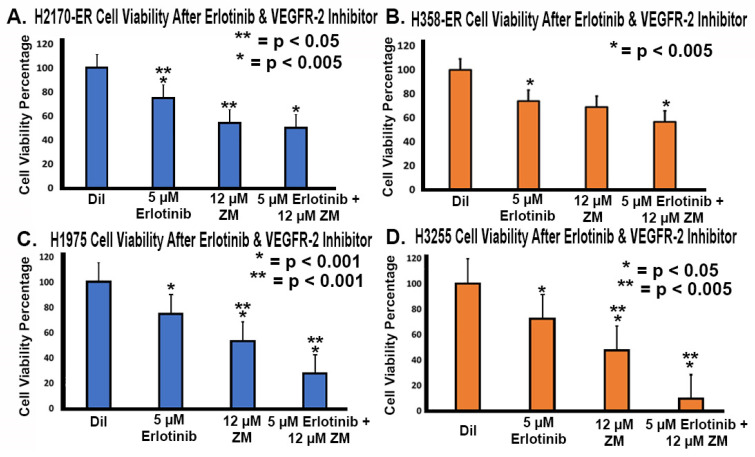
**Effect of anti-VEGFR-2 treatments in resistant and mutated NSCLC cell lines.** A total of 8000 H2170-ER, H358-ER, H1975, and H3255 NSCLC cells were treated with diluent (control), 5 µM Erlotinib treatment, 12 µM ZM treatment, and a combination of 5 µM Erlotinib and 12 µM ZM. Cell viability was assessed with an MTT cell viability assay to study the efficacy of Erlotinib in conjunction with ZM, a VEGFR-2 inhibitor, relative to the control. In (**A**) H2170-ER cells, (**B**) H358-ER cells, **(C**) H1975-ER cells, and (**D**) H3255-ER cells, percent cell viability was measured. Six duplicates from each cell line were used for each treatment group. Absorbance was recorded and percent viability was calculated using appropriate controls for each sample. Statistical analysis was performed, and the results were found to be statistically significant (* = *p* < 0.05) by way of two-tailed *t*-test analysis. All treatment groups showed statistically significant results compared to the diluent. Treatment groups without an asterisk (*) did not show statistically significant results. One asterisk (*) denotes statistical significance in all the bars with one asterisk, two asterisks (**) suggest statistical significance between all the bars with two asterisks and three asterisks (***) suggests that the data is significant in comparison to both one and two asterisks.

After studying the effects of treatment against VEGF, we studied the inhibition of VEGFR-2 in Erlotinib-resistant and EGFR-mutated NSCLC cell lines to further elucidate its role in EGFR-TKI resistance. An MTT cell viability assay was performed with 5 µM Erlotinib treatment, 12 µM ZM 323–881 HCl (ZM) (VEGFR-2 inhibitor) treatment, and a combination treatment of Erlotinib and ZM. We tried different concentrations of ZM and selected a concentration of ZM which resulted in less than 50% inhibition. We used Erlotinib at a concentration of 5 uM, since the resistant cell lines studied showed 25–26% inhibition with 5 μM Erlotinib. We wanted to study a combination effect, so we kept the concentration of Erlotinib at half the dose we used for other aspects of the studies. As seen in Figure 7, we observed that 5 µM Erlotinib treatment reduced cell viability to between 72% and 75%. We also observed that 12 µM ZM treatment reduced cell viability to between 47% and 70% (Figure 7). With a combination of Erlotinib (5 µM) and ZM (12 µM), cell viability was reduced to between 9% and 57% (Figure 7). There was a significant decrease in cell viability with a combination of Erlotinib (5 µM) and ZM (12 µM) as compared to Erlotinib and ZM alone in double-mutant H1975 cells and single-mutant H3255 cells, whereas in H2170ER and H358ER cells ZM minimally enhanced the efficacy of erlotinib. However, decreased viability was observed with ZM when compared with Erlotinib alone in H2170ER cells, indicating that ZM is effective in EGFR mutant cell lines.

### 3.8. VEGFR-2 Expression in Late-Stage NSCLC Tumor Samples with Kaplan–Meier Analysis

**Figure 8:**
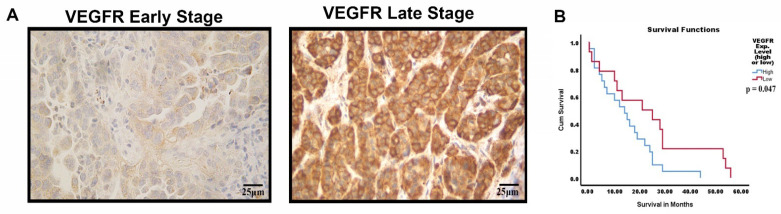
Detection of VEGFR-2 in NSCLC tumor sections via IHC, and survival analysis for patients with high/low VEGFR-2 expression. (**A**) NSCLC tumor sections were stained for VEGFR-2 expression (brown). Tumor sections in early and late stages of NSCLC with low and high expression of VEGFR-2 localized in the nucleus and in the cytoplasm. (**B**) Kaplan–Meier survival analysis of late-stage NSCLC patients demonstrated a difference in survival time from date of diagnosis in patients with high vs. low expression of VEGFR-2. The equality of survival distributions for the two expression groups was assessed by way of a log-rank test with *p* < 0.05 indicating a significant difference in survival.

Immunohistochemistry (IHC) was performed to analyze the expression of VEGFR-2 in tumor tissues from NSCLC patients. Forty-eight NSCLC tumor tissues were assessed for VEGFR-2 expression through IHC with VEGFR-2 staining (Figure 8A). Kaplan–Meier survival analysis of NSCLC patients was performed to correlate VEGFR-2 expression with months of survival after prognosis (Figure 8B). Our results demonstrated a shorter median survival time after prognosis in late-stage NSCLC patients with high levels of VEGFR-2 expression (14 months) compared to late-stage NSCLC patients with low VEGFR-2 expression (21 months) (*p* < 0.05) (Figure 8B).

## 4. Discussion

In this study, we recorded upregulation of the gene expression of VEGF, VEGFR-2, and NP-1 in Erlotinib-resistant cell lines as compared to parental cells and in EGFR double-mutant cells as compared to their single-mutant counterparts (Figure 1 and Figure 2). In all the cell lines studied, the presence of Erlotinib resulted in VEGFR-2 and NP-1 downregulation compared to the diluent (Figure 2A). These results suggest that Erlotinib has an inhibitory effect on VEGFR-2 and NP-1 protein expressions. Furthermore, the presence of EGF was tied to increased VEGFR-2 and NP-1 expressions, demonstrating the potential for EGF to stimulate the release of angiogenic markers, such as VEGF, which causes VEGFR-2 and NP-1 protein levels to increase [40]. Earlier studies have also shown that EGF increases NP-1 production and mRNA expression of VEGFRs, resulting in increased affinity of VEGF isoforms to VEGFR-2 [40].

**Figure 9:**
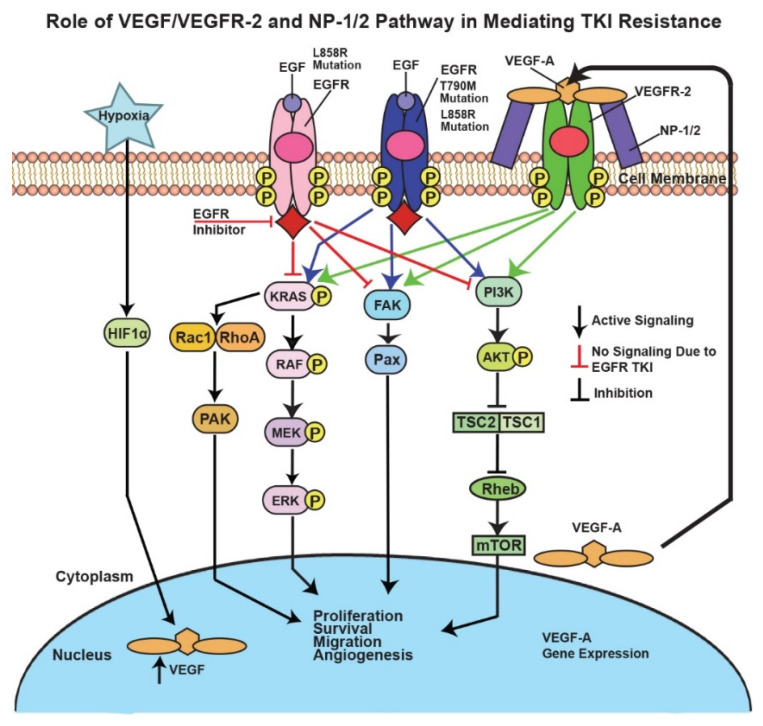
**Mechanisms of resistance to molecularly targeted therapy in NSCLC**. In TKI-resistant NSCLC cells activated EGFR leads to activation of the MAPK, FAK-Paxillin, and PI3K-AKT-mTOR downstream pathways, resulting in increased proliferation, survival, migration, and angiogenesis. EGFR TKIs inhibit this activation; however, due to mutations (primary L858R and secondary T790M mutations), tumor cells acquire resistance against these TKIs, activating these pathways further. Hypoxia also induces upregulation of VEGF. Upregulation of VEGF, VEGFR-2, and NP-1/2 may also lead to activation of the downstream pathways mentioned above, causing TKI resistance in NSCLC.

VEGFR-2 and NP-1 protein expressions were also increased in EGFR double-mutant H1975 cells compared to H2170-ER and H358-ER cell lines, a result attributed to the T790M mutation in H1975 cells (Figure 2B). Unlike H2170 cells, H358 cells possess the KRAS mutation, a GTP-bound protein mutation which results in over-activation of the MEK-ERK and PI3K-AKT angiogenic pathways [41]. Gefitinib and Erlotinib (first-generation TKIs) exhibited no efficacy in lung adenocarcinoma patients with KRAS mutations, which could have been due to downregulation of VEGFR-2 in H2170-ER cells compared to H358-ER cells when both cell lines were exposed to Erlotinib (Figure 2) [42]. H1975 cells demonstrated an even larger fold change in VEGFR-2 and NP-1 protein levels after Erlotinib exposure as compared to H3255 cells (Figure 2b), which may have been due to the presence of both L858R and T790M mutations. The L858R EGFR mutation is the most sensitizing oncogenic point mutation [43]. The T790M mutation causes resistance to TKIs by way of steric hindrance, which interrupts TKI binding to the ATP binding pocket [44]. In cells with the T790M mutation in EGFR, there is a high affinity for ATP, a competitor of Erlotinib. This prevents Erlotinib from binding to EGFR and blocking its downstream action, so EGFR is still active. Studies have found that patients with both T790M and L858R mutations showed a drastic increase in EGFR phosphorylation compared to patients with only the L858R mutation [45]. The increase in EGFR kinase activity observed in this study further links T790M mutation to tumor angiogenesis [46]. Previous studies have also tied KRAS mutations and EGFR-activating mutations (L858R and T790M) to angiogenesis in NSCLC [46,47]. Our studies suggest that these mutations may also result in increased VEGFR-2 and NP-1 protein expression, thereby causing sustained angiogenesis in the presence of Erlotinib (Figure 9).

These mutations cause altered expression of EGFR, which results in the activation of downstream signaling pathways, such as the MAPK, FAK/Paxillin, and PI3K/AKT/mTOR pathways, which, in turn, promotes tumor cell proliferation, survival, migration, and angiogenesis [48].According to flow cytometry results, we observed statistically significant increases in VEGFR-2 and NP-1 protein levels on the cell surfaces of EGFR-TKI-resistant cells compared to their corresponding TKI-sensitive cell lines (Figure 3). Our results also demonstrated increased cell surface expression of NP-1 in Erlotinib-resistant and EGFR double-mutated cells compared to parental and single-mutated cells, respectively (Figure 3). This novel observation has not been reported previously. IF results also demonstrated increased VEGFR-2 and NP-1 expression in H358-ER and H1975 cells compared to their parental and single-mutant counterparts, H358-P and H3255, respectively (Figure 4). These results may be explained by decreased internal trafficking and recycling of NP-1.

Studies have shown that NP-1 interacts with VEGFR-2 in a cis position, which, in turn, increases the binding affinity of VEGF to VEGFR-2 [49]. In this cis NP-1/VEGFR-2 position, where NP-1 and VEGFR-2 are expressed on the same cell and form a heterocomplex induced by VEGF, VEGFR-2 is internalized at a rapid rate [33,49]. In the trans NP-1/VEGFR-2 position, where NP-1 and VEGFR-2 are instead expressed on adjacent cells, the binding of VEGF to the NP-1/VEGFR-2 complex slows the internalization of VEGFR-2 on the cell surface, suppressing angiogenesis [33,49]. Studies have also shown that NP-1 increases VEGF signaling by promoting VEGFR-2 clustering or endocytosis [50]. In addition to ligand-induced endocytosis, VEGFR-2 can also undergo constitutive endocytosis in the absence of VEGF [51]. Studies suggest that VEGFR-2 undergoes constitutive endocytosis to protect the receptor from plasma membrane cleavage, thus preserving VEGFR-2 functional ability until VEGF-induced activation [52]. Our results demonstrated decreased membranous expression of VEGFR-2 in H358-P and H3255 cell lines but sustained VEGFR-2 expression in the cytoplasm and nucleus (Figure 3 and Figure 4). Increased membranous, cytoplasmic, and nuclear expressions of VEGFR-2 and NP-1 were observed in H358-ER and H1975 cells, suggesting the formation of a cis heterodimer complex, which may result in angiogenesis (Figure 4). The increased cytoplasmic expression of VEGFR-2 that we observed in H358-ER and H1975 cells could have been due to KRAS and EGFR mutations, which may increase the rate of constitutive endocytosis to protect against shedding, therefore keeping VEGFR-2 in a functional state ready for VEGF binding. The increased membranous and internal localization of these angiogenic receptors suggests that these mutations may play a key role in EGFR-TKI resistance.

Our results also demonstrated that total VEGF secretion increased over time (Figure 5). VEGF secretion does not appear to be a major factor in EGFR-TKI resistance, since there was a minimal difference in VEGF secretion levels across the ER and double-mutant cell lines studied. MTT results further support this inference. As seen in Figure 6, treatment with Avastin alone showed a minimal difference when compared to treatment with Erlotinib alone, and treatment with a combination of Erlotinib and Avastin did not yield an additive effect in Erlotinib-resistant H2170 and H358 cell lines. Although additive effects of Avastin and Erlotinib were observed in H1975 and H3255 cells, there was lower cell viability in H3255 compared to H1975 cells since H1975 has a T790M mutation which confers resistance to Erlotinib. Avastin did not have an additive effect in H2170-ER and H358-ER cells when used in combination with Erlotinib because both treatments may be acting on the same downstream pathways, such as the PI3K-AKT-mTOR or MAPK pathways. This experiment confirms our earlier results, which showed that VEGF secretion may not play a major role in these EGFR-TKI-resistant cell lines, since inhibiting VEGF in the presence of Erlotinib had no significant result.

After determining that VEGF inhibition is not useful for overcoming EGFR-TKI resistance, we inhibited VEGFR-2 using a selective VEGFR-2 inhibitor, ZM 323–881 HCl (ZM). Based on our results, treatment with ZM alone and treatment with a combination of Erlotinib and ZM both significantly decreased cell viability in Erlotinib-resistant and EGFR-mutated cell lines (Figure 7). These results are promising and suggest that the L858R and T790M EGFR mutations are sensitive to combinatory treatment. The additive effect of ZM used in combination with Erlotinib in the H3255 and H1975 cell lines suggests that combined inhibition of the VEGFR-2 and EGFR pathways may be an effective treatment method in NSCLC patients with EGFR mutations. In the H2170-ER cell line, in which an additive effect was not observed, ZM as a standalone treatment was still shown to be effective as compared to Erlotinib (Figure 7). In the H358-ER cell line, however, the KRAS mutation demonstrated greater resistance than the EGFR mutation. Since KRAS mutations affect 20–30% of NSCLC patients and their presence is often associated with shorter overall patient survival compared to EGFR mutations, studies suggest that the KRAS mutation is a negative predictor for EGFR-TKI treatments [53,54]. In our investigation, the KRAS mutation demonstrated a role in EGFR-TKI resistance, since it resulted in increased gene and protein expressions as well as increased cell surface expression of the angiogenic biomarkers VEGFR-2 and NP-1 (Figure 1, Figure 2, Figure 3 and Figure 4). Therefore, we believe that the targeting of RAS or its upstream signaling proteins in patients with a KRAS mutation could be a valuable area of study for overcoming EGFR-TKI resistance. Furthermore, with increased knowledge of the type of mutations a patient may present, proper treatment options based on mutational analysis may be provide effective means to overcome EGFR-TKI resistance.

During NSCLC tumor screening with VEGFR-2 and NP-1 by IHC assays, we studied the expression levels of these angiogenic biomarkers in late- and early-stage NSCLC patients. VEGFR-2 is a transmembrane tyrosine kinase receptor, and studies have shown that VEGFR-2 is internalized with the help of NP-1 [49]. Our flow cytometry results revealed that VEGFR-2 is present on the cell surface of NSCLC tumor cells and IHC results showed expression in the cytoplasm and nuclear compartments of NSCLC patients’ cells (Figure 3 and Figure 8) [55,56]. VEGFR-2 can thus be internalized and translocated to both the cytoplasm and the nucleus, independent of high or low expression [55,56]. Our results showed NP-1 to be clearly expressed in the cytoplasm but minimally expressed in the nucleus (Figure 3 and Figure 4). Lastly, we wanted to study whether VEGFR-2 can be utilized as a prognostic marker in NSCLC patients. Using Kaplan–Meier analysis, late-stage NSCLC patients with high expression of VEGFR-2 were found to have a median survival time of 14 months, while late-stage NSCLC patients with low expression had a median survival time of 21 months (Figure 8). Therefore, high expression of VEGFR-2 appears to be associated with poor prognosis in patients with NSCLC. In a clinical setting, the utilization of VEGFR-2 as a prognostic marker could help physicians devise an appropriate care plan based on VEGFR-2 expression levels in patients.

## 5. Conclusions

This study investigated three angiogenic biomarkers, VEGF, VEGFR-2, and NP-1, that may play a role in EGFR-TKI resistance. In EGFR-TKI-resistant cells compared to TKI-sensitive cell lines, an upregulation of VEGFR-2 and NP-1 gene and protein expression, as well as increased cell surface expression, was observed. In addition, we observed that combinatorial treatment of Erlotinib and a selective VEGFR-2 inhibitor may serve as a more effective treatment option than inhibition of VEGF with a humanized monoclonal antibody Avastin. TKIs such as Erlotinib and Gefitinib have no effect on lung adenocarcinoma patients with KRAS mutations; however, combination therapy with Erlotinib and VEGFR-2 inhibitor can help achieve better prognoses for these patients. Our studies suggested that high VEGFR-2 expression is associated with poor prognosis in NSCLC patients, and we also studied single- and double-mutant NSCLC cell lines to provide a new outlook on ways to overcome EGFR-TKI resistance in NSCLC patients.

## Figures and Tables

**Table 1:** qPCR primers used for expression analysis.

Gene	Sequence	Melting Point	DNA Bases
VEGF	F: 5′ CGCAAGCTTAGGAGTACCCTGATGAG 3′	60.7 °C	26
	R: 5′ CCGTCTAGAACATTTGTTGTGCTGT 3′	57.6 °C	25
VEGFR-2	F: 5′ GCAGGGGACAGAGGGACTTG 3′	60.1 °C	20
	R: 5′ GAGGCCATCGCTGCACTCA 3′	60.4 °C	19
NP-1	F: 5′ ATGGAGAGGGGGCTGCCG 3′	63.0 °C	18
	R: 5′ CTATCGCGCTGTCGGTGTA 3′	56.9 °C	19
GAPDH	F: 5′ ATGACATCAAGAAGGTGGTG 3′	54.4 °C	20
	R: 5′ CAGGAAATGAGCTTGACAAA 3′	55.8 °C	20

## Supplementary Materials

The following supporting information can be downloaded at: https://www.mdpi.com/article/10.3390/cells11101694/s1, Table S1. Table showing doubling times of H2170 and H358 parental and resistant cell lines. Table S2. Table showing demographics for NSCLC patients used for studying expression of VEGFR-2.
