# Supplementary material for: Mediating EGFR-TKI Resistance by VEGF/VEGFR Autocrine Pathway in Non-Small Cell Lung Cancer
Source: Cells. 2022 May 19;11(10):1694. doi: 10.3390/cells11101694 (PMC9139342; doi:10.3390/cells11101694)
Supplement: Supplementary file 1 [file cells-11-01694-s001.zip › cells-1415161-supplementary.pdf]

TABLE S1. Table showing doubling times of H2170 and H358 parental and resistant cell lines.

Cell Line	No of Cells Plated	No of Cells Counted	Average	No of Days	Doubling Time (Days)	Average Doubling Time	Standard Deviation
H2170P	1×10^5	1.34×10^6	1.39×10^6	6	1.6	1.58	0.017
H2170P	1×10^5	1.42×10^6		6	1.57		
H2170P	1×10^5	1.42×10^6		6	1.57		
H2170ER	1×10^5	1.23×10^6	1.26×10^6	6	1.82	1.8	0.062
H2170ER	1×10^5	1.19×10^6		6	1.85		
H2170ER	1×10^5	1.38×10^6		6	1.73		
H358P	1×10^5	7.8×10^5	7.6×10^5	8	2.7	2.74	0.047
H358P	1×10^5	7.3×10^5		8	2.79		
H358P	1×10^5	7.7×10^5		8	2.72		
H358ER	1×10^5	1.25×10^6	1.17×10^6	8	2.41	2.48	0.061
H358ER	1×10^5	1.15×10^6		8	2.49		
H358ER	1×10^5	1.12×10^6		8	2.53		

TABLE S2. Table showing demographics for NSCLC patients used for studying expression of VEGFR-2.

Gender		Race		Smoking Status		Age
Male	Female	Caucasian	Non-Caucasian	<30 pack years	>30 pack years	
25	23	46	2	9	37	42-89
